# Growth performance, morphometric of the small intestine, lymphoid organ, and ovary of laying hens supplemented with Dates (*Phoenix dactylifera* L.) extract in drinking water

**DOI:** 10.14202/vetworld.2022.350-359

**Published:** 2022-02-16

**Authors:** L. U. Albab, T. I. Claudya, R. Oktafianti, N. Salsabila, R. D. Putri, H. T. S. S. G. Saragih

**Affiliations:** 1 Post Graduate Program of Biology, Department of Tropical Biology, Universitas Gadjah Mada, Yogyakarta, Indonesia; 2 Graduate Program of Biology, Department of Tropical Biology, Universitas Gadjah Mada, Yogyakarta, Indonesia; 3 Laboratory of Animal Development Structure, Faculty of Biology, Universitas Gadjah Mada, Jl. Teknika Selatan, Sekip Utara, Mlati, Sleman, Yogyakarta 55281, Indonesia

**Keywords:** dates water extract, feed additive, ovary, performance, small intestine

## Abstract

**Background and Aim:**

Antibiotic, improves the growth performance of laying hens when used as a feed additive; however, it has been banned in Europe. Furthermore, secondary metabolites used as a substitute for antibiotics are compounds produced by plants. Therefore, this aims to determine the effect of dates water extract (DWE) on the performance of laying hens. This study used dates containing secondary metabolites as a feed additive and substitute for antibiotics.

**Materials and Methods:**

A completely randomized design was used, dividing 400 Lohmann brown day old chick into five groups (each group has five replications and each replication consisted of 16 laying hens). Furthermore, there were two control groups such as mineral water control group and antibiotic growth promoters (basal feed+50 mg/kg of bacitracin), and three DWE groups such as 5% DWE (50 mg/mL), 10% DWE (100 mg/mL), and 20% DWE (200 mg/mL). Dates extract treatment was administered through drinking water for 54 days, whereby three laying hens from each replication were taken randomly and decapitated on the neck. Afterwards, a necropsy was performed for histological preparations of the small intestine, ovary, and lymphoid organs. The structure and morphology of the small intestine, and ovaries were observed through histological preparations, while lymphoid organs were observed through histological preparation and morphometry, and body morphometry, body weight, feed intake and weight gain were observed by measurements and weighing.

**Results:**

Small intestine morphology, ovarian follicle, and growth performance of the DWE2 group increased significantly compared to the control group, but the lymphoid organs index was influenced by DWE1.

**Conclusion:**

The administration of 10% dates extract (100 mg/mL) in drinking water improves the morphology of the small intestine, ovarian follicles, lymphoid organs, and growth performance.

## Introduction

High growth rate and feed efficiency are the two main goals of the poultry industry. Furthermore, one factor that influences this goal is a healthy digestive system. Laying hens intestinal mucosa plays an essential role in providing a barrier between the internal tissues and the content that penetrates the body [1]. In addition, poultry nutrition is linked to the hypothalamic–pituitary–gonadal axis [2]. Therefore, a healthy digestive system increases laying hen’s growth rate and the maturation process of the reproductive organs. Egg production performance is also related to environmental conditions, feeding management, and follicular development [3]. Antibiotic growth promoters (AGP) are used to maintain a healthy intestinal environment and improve the growth performance of livestock. However, Europe has banned the use of AGP compounds in poultry diets. At the same time, North America anticipates its reduction in poultry by searching for other components as alternatives to improve growth performance and maintain animal health. This component includes phytogenic feed additives, which improve the performance of laying hens by providing a healthy digestive system [4]. Meanwhile, herbal extracts such as date palm have been used as feed additives for several years due to the European Union’s ban on antibiotics in animal feed [5,6]. Although dates contain fiber, sugar, protein, vitamins, and minerals, they also comprise phenols (p-coumaric acid, ferulic acid, catechin, gallic acid, chlorogenic acid, and syringic acid) flavonoids [7]. Its lignins and tannins also contain high antioxidant and antimicrobial activity [8].

A previous study using date palm kernel (DPK) as a feed additive showed that the addition of 3-4% DPK in poultry feed significantly decreased feed intake and increased water intake, body weight, weight gain, and carcass weight [9]. The addition of 2% and 4% date seeds to laying hens feed improves the health profile, which is observed in the increase in body weight, organ weight, antibody titer, Interferon-ɣ, interleukin-2, and antioxidant status of laying hens compared to the group with mannan-oligosaccharides and β-glucans [10].

Based on studies that have been conducted by Tareen *et al*. [9] and El-far [10]; it has been concluded that dates potentially increase the performance of laying hens growth. However, investigation using dates which are generally in the form of a powder or crude extract as a feed additive has limited information concerning the effects of dates water extract (DWE) on the histological structures and performance of laying hens.

Therefore, this study aims to determine the potential of DWE as a natural feed additive in improving the performance of laying hens.

## Materials and Methods

### Ethical approval

The study was approved by the Ethical Board of the Faculty of Veterinary Medicine, Gadjah Mada University, with certification number 0022/EC-FKH/EKS/2019.

### Study period and location

The study was conducted from May to August 2019 in Sawit Sari Research Station and Laboratory of Animal Development Structure, Faculty of Biology, Universitas Gadjah Mada, Indonesia.

### Date palm water extract

This study used Khenaizi dates of Date Crown brand from Al Foah LCC, United Arab Emirates, in which the seeds were collected and weighed to determine the concentration. Dates weighing 5 g, 10 g, and 20 g were immersed in 100 mL warm water at 37°C in a glass bottle and left overnight in the refrigerator at 4°C. Afterward, the water extract solution was filtered to remove dates residue [11].

### Experimental design

Four hundred 1-day-old chicks of Lohmann Brown laying hens were purchased from Japfa Comfeed Indonesia. The birds were randomly divided into five groups with five replications in each group, consisting of 16 laying hens each. DWE was provided through drinking water, where day-old chick in the control group (negative control) was administered basal feed and mineral water, the AGP group was administered basal feed+50 mg/kg of bacitracin and mineral water, DWE1 was administered 5% DWE (50 mg/mL), DWE2 was administered 10% DWE (100 mg/mL), and DWE3 was administered 20% DWE (200 mg/mL). The basal feed formulated by Sari Rosa Asih Feedmill Inc. is listed in Table-1 [12]. Acclimatization was performed from 0 to the 3^rd^ day, while the treatment started from the 4^th^ to the 54^th^ day. During the acclimatization process, all chicken were given mineral water *ad libitum*. The ambient temperature was measured twice a day, and DWE was administered for a half-day in the morning, the water intake data are shown in Table-2. Dates extract treatment was administered through drinking water changed into mineral water in the afternoon and provided in *ad libitum*. The cages were cleaned every 3 days, and the measurement of the body weight was conducted every 6 days starting from 0 to the 54^th^ day, while morphometry was measured on 0 and 54^th^ days.

**Table 1:** Basal feed formulation and nutrition content.

Composition of feed	Starter (%)	Grower (%)
Corn	60	60.92
Soybean meal	29	21
Rice bran	-	11.8
Meat bone meal	7	3
Crude palm oil	3.0	1.25
Dicalcium phosphate	0.04	0.8
Limestone	-	0.42
Salt	0.12	0.25
Sodium bicarbonate	0.25	-
Lysine HCL	0.15	-
Dl-Methionine	0.29	0.03
Threonine	-	0.02
Vitamin	0.04	-
Mineral	0.05	-
Premix Vitamin Mineral^a^	-	0.5
Phytase	0.01	0.01
Coccidiostat	0.05	-
Total	100	100
Calculated composition^b^	
Dry matter (%)	88.91	99
Metabolizable energy (MJ)	11.88	11.46
Crude protein (%)	22.174	17.475
Crude fat (%)	2.582	3.011
Crude fiber (%)	2.100	3.471
Dig. Lysine (%)	1.104	0.811
Dig. Methionine (%)	0.569	0.297
Dig. Methionine+cysteine (%)	0.834	0.521
Calcium (%)	0.808	0.851
Phosphorus, total (%)	0.735	0.933
Phosphorus, available (%)	0.605	0.556

a=Premix vitamin mineral provided the following per kilogram of diet (Vitamin A=15000 IU, Vitamin D_3_=3000 IU, Vitamin E=22.5 mg, Vitamin K_3_=3 mg, Vitamin B_1_=3 mg, Vitamin B_2_=9 mg, Vitamin B_6_=4.5 mg, Vitamin B_12_=30 mcg, biotin=30 mcg, folic acid=1.5 mg, niacin=45 mg, pantothenic acid=1.5 mg, Vitamin C=0 mg, choline=2090 mg & 1242 mg); Premix mineral provided the following per kilogram of diet (Cu=12 mg, Fe=72 mg, Iodine=0.9 mg, Mn=84 mg, Se=0.3 mg, Zn=60 mg); b=Proximate, amino acids, minerals, and metabolizable energy were obtained from calculated values[12]

**Table 2:** Water intake (mL) Lohmann Brown’s chicken for a ½ day/bird.

Week	Treatment				

	Control	AGP	DWE1	DWE2	DWE3
1	138.06±8.599	129.61±14.626	142.17±15.161	149.89±16.934	156.89±22.937
2	185.05±13.836	179.43±14.66	198.738±9.993	192.33±13.762	187.929±14.050
3	280.50±10.030	277.12±7.943	280.93±11.575	283.452±8.852	281.286±8.246
4	466.31±17.300	447.64±14.438	463.17±17.018	460.07±15.912	446.59±14.959
5	497.52±1.677	494.33±3.252	500.00±0.000	497.67±1.517	495.714±2.261
6	667.62±14.322	675.71±16.014	649.81±18.921	673.09±12.831	669.26±13.558
7	683.71±8.808	672.62±15.417	663.12±13.096	657.548±8.950	668.548±8.905
8	728.57±18.443	727.62±18.711	727.38±18.785	697.05±29.991	720.05±21.485

Control=Mineral water; AGP=Bacitracin 50 mg/kg basal diet+mineral water; DWE1=5% dates water extract; DWE2=10% dates water extract; DWE3=20% dates water extract, dates water extract has been given through drinking water

### Euthanasia and organ preparation

On day 54^th^, old laying hens fasted for 6 h, and three laying hens from each replication were chosen randomly and decapitated on the neck. Afterward, they were dissected on the ventral using scissors and scalpels. The small intestine, ovary, and lymphoid organs were washed with normal saline (NaCl 0.9%), then the small intestine was separated between the duodenum, jejunum, and ileum [13,14]. Furthermore, the lymphoid organs were measured by analytical weight, and the index was calculated by the following formula:


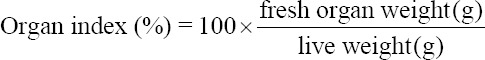


### Histological preparation of small intestine, ovary, and lymphoid organ

A slide set of the small intestine, ovarian, spleen, and bursa of Fabricius histology was made using the paraffin method [3]. First, the samples were fixed with Bouin solution for 12 h, after which the small intestine slides were stained with Periodic acid Schiff-alcian blue (PAS-AB; Merck, Darmstadt, Germany) to measure villi length, crypt depth, and the number and area of goblet cells. The PAS reagent was used to detect neutral mucus produced by goblet cells, while the AB reagent was used to detect acid mucus [15-17]. In addition, the ovaries slides were stained with hematoxylin-eosin to detect cytoplasm and cell nuclei. In contrast, other slides were stained with immunohistochemistry method using Starr trek universal Horseradish peroxidase-detection system (Biocare Medical, USA) with modified anti-proliferating cell nuclear antigen (PCNA; Abcam, USA) to detect cell proliferation. The spleen and bursa of Fabricius slides were stained with hematox­ylin-eosin (Merck) to detect the spleen and bursa of Fabricius’s white pulp and follicle area, respectively.

### Small intestine morphology

The small intestine histology was observed using Leica DM750 (Leica Microsystems, Germany) with a magnification of 4×0.10. Furthermore, the Villi length, crypt depth, and goblet cell area were measured using the Image J 1.52 ver­sion (NIH, USA). The observation of the small intestine consists of several stages, namely:

### Measurement of length and area of villi

The length, basal width, and apical width villi in the duodenum, jejunum, and ileum were measured using Image J software (NIH) with five fields of view on each slide. Furthermore, the villi area (mm^2^) was calculated using the following formula from Setiawan *et al*. [17]:


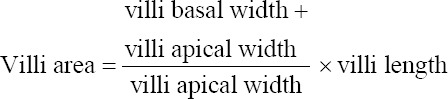


### Crypt depth measurement

The crypt depth was observed in five fields of view on each histological slide, while the ratio between the villi length and crypt depth was calculated using the following formula from Saragih *et al*. [18]:


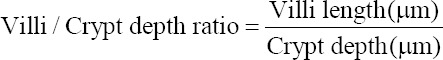


### Calculation of the number and area of goblet cells

The number and area of goblet cells were calculated using a magnification of 10×0.25. The number of goblet cells was observed by counting the goblet cells in the small intestinal villi along 500μm, while the goblet cells area were measured from the edge of the membrane surrounding the goblet cells (cup) on a cross-section of the villi using Image J software (NIH) [17].

### Ovaries observation

Follicles were counted serially based on the type, including primordial, primary, secondary, and de Graaf follicles. Furthermore, the calculated follicles consist of clear nuclear cells and are traced to the same position on the previous coupes to avoid biased calculations [19].

### Chicken morphometry

Chicken morphometry with variables consisting of back length, chest circumference, depth, and shank length and circumference was measured at the 0 and 54^th^ day using midline and caliper. The chest was measured using a caliper, while other variables were measured using the midline [13,20].

### Calculation of feed conversion ratio (FCR)

Feed consumption was calculated daily, from the 4^th^ to the 54^th^ day. Furthermore, the feed consumption ratio is the ratio between feed intake and weight gain which was calculated by the following formula:


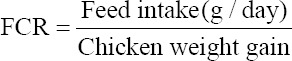


### Statistical analysis

The collected data were analyzed using one-way analysis of variance and presented as mean±standard error of the mean, followed by Duncan’s test with a significance level of 5% (p≤0.05). All data were analyzed using the Statistical Package for the Social Sciences (SPSS 25.0) computer program (SPSS, Inc., Chicago, IL, USA) for windows.

## Results

### Small intestine morphology

The small intestine morphology of 54 days old laying hens, which were administered DWE through drinking water, is shown in Table-3. The length of the duodenal villi in the DWE2 and DWE3 groups was higher than the control and AGP groups. Furthermore, the duodenum’s crypt depth and the villi area were not significantly different. However, the highest crypt depth was in the DWE2 group, and the highest villi area was in the DWE3 group. The duodenal villi/crypt ratio in the DWE3 group was significantly (p≤0.05) higher than the control and AGP groups. DWE significantly increased the number and area of goblet cells.

**Table 3:** Small intestine morphology of 54 days old laying hens, Lohmann Brown Strain, treated with dates (*Phoenix dactylifera* L.) water extract.

Variables	Treatments				

	Control	AGP	DWE1	DWE2	DWE3
Duodenum				
Villi Length (mm)	1.12±0.047^a^	1.16±0.015^a^	1.13±0.044^a^	1.34±0.040^b^	1.29±0.623^b^
Crypt Depth (mm)	0.24±0.014	0.26±0.020	0.26±0.022	0.28±0.005	0.24±0.022
Villi/Crypt Rasio	3.98±0.223^a^	4.86±0.422^ab^	4.73±0.366^ab^	5.06±0.195^ab^	5.92±1.640^b^
Villi Area (mm^2^)	3.78±0.452	4.13±0.237	3.76±0.400	4.24±0.394	4.59±0.487
Number of Goblet Cells	108.53±5.258^a^	131.66±13.914^ab^	140.57±8.771^b^	210.75±8.589^c^	198.45±9.367^c^
Goblet cells Area (μm^2^)	33.71±3.344^a^	49.63±5.265^b^	33.86±4.665^a^	61.12±2.357^b^	50.28±3.198^b^
Jejunum				
Villi Length (mm)	0.90±0.084^a^	1.03±0.104^ab^	0.96±0.026^ab^	1.17±0.096^b^	1.03±0.028^ab^
Crypt Depth (mm)	0.24±0.022	0.25±0.013	0.25±0.016	0.26±0.023	0.24±0.012
Villi/Crypt Rasio	3.98±0.497	4.21±0.393	4.07±0.317	5.09±0.762	4.74±0.195
Villi Area (mm^2^)	2.75±0.328	3.09±0.385	2.83±0.176	3.67±0.278	3.07±0.190
Number of Goblet Cells	120.05±6.354^a^	153.23±7.363^bc^	147.26±14.112^ab^	224.01±14.795^d^	180.95±7.120^c^
Goblet cells Area (μm^2^)	26.62±1.516^a^	32.94±1.313^a^	34.74±3.218^ab^	44.64±3.486^b^	44.53±5.135^b^
Ileum				
Villi Length (mm)	0.72±0.048	0.87±0.090	0.82±0.028	0.86±0.038	0.95±0.015
Crypt Depth (mm)	0.17±0.004	0.17±0.009	0.19±0.015	0.19±0.022	0.19±0.021
Villi/Crypt Rasio	4.05±0.186^a^	5.44±0.685^ab^	4.70±0.374^ab^	5.21±0.522^ab^	6.36±0.853^b^
Villi Area (mm^2^)	2.04±0.147^a^	2.47±0.228^ab^	2.28±0.017^ab^	2.33±0.168^ab^	2.66±0.096^b^
Number of Goblet Cells	97.51±4.265^a^	141.08±5.268^b^	149.51±10.289^b^	180.47±4.835^c^	190.54±7.454^c^
Goblet cells Area (μm^2^)	17.28±0.745^a^	30.14±1.77^bc^	25.63±2.337^ab^	47.75±1.461^d^	35.59±5.430^c^

Control=Mineral water; AGP=Bacitracin 50mg/kg basal diet+mineral water; DWE1=5% dates water extract; DWE2=10% dates water extract; DWE3=20% dates water extract, dates water extract has been given through drinking water.

^a-d^ Different letters on values in the same row indicate a significant difference (p≤0.05)

**Figure-1:**
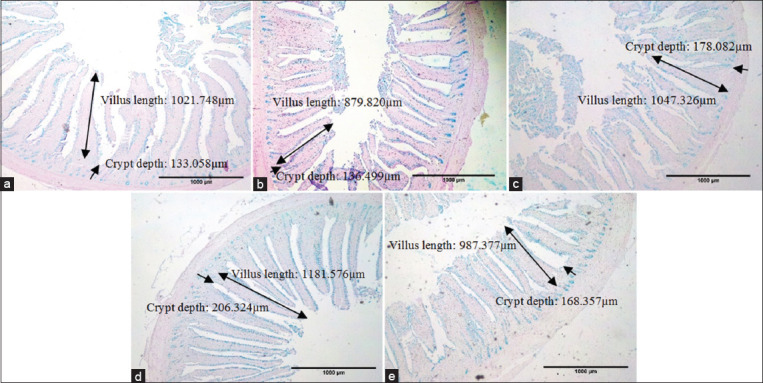
Jejunum morphology of 54 days old Lohmann Brown Strain treated with dates water extract using 4×0.10 magnification. (a) Control; (b). AGP group; (c) DWE1 group; (d). DWE2 group; (e). DWE3 group. Control=Mineral water; AGP=Bacitracin 50 mg/kg basal diet+mineral water; DWE1=5% dates water extract; DWE2=10% dates water extract; DWE3=20% dates water extract, dates water extract has been given through drinking water ⟷=villus length, ⟶=Crypt depth. This figure shows that the DWE2 group had higher villi and crypt depth compared to other groups.

The jejunal morphology of laying hens showed that the DWE2 group was significant (p≤0.05). In addition, DWE positively affected the crypt depth, villi area, and villi/crypt ratio of jejunum. Figure-1 shows the jejunum morphology of laying hens. The number of goblet cells in the DWE2 group was significantly higher than in other groups. Meanwhile, the goblet cell area in the DWE2 and DWE3 groups was significantly more significant than the control and AGP groups.

The effect of administering DWE on the ileum morphology of laying hens positively affected villi length and crypt depth. The DWE3 group significantly increased the villi area and the ratio of villi/crypt of the ileum. Futhermore, the DWE2 and DWE3 groups significantly increased the number of goblet cells (p≤0.05), while the DWE2 groups increased the goblet cells area.

### Ovarian morphology

**Figure-2:**
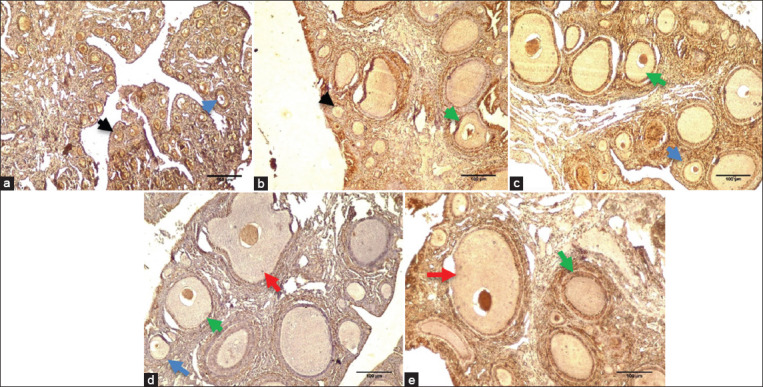
Ovarian morphology of 54 days old Lohmann Brown Strain treated with dates water extract using 10×10 magnification. (a) Control; (b). AGP group; (c). DWE1 group; (d). DWE2 group; (e). DWE3 group. Control=Mineral water; AGP=Bacitracin 50 mg/kg basal diet+mineral water; DWE1=5% dates water extract; DWE2=10% dates water extract; DWE3=20% dates water extract, dates water extract has been given through drinking water. Black arrow=Primordial follicle; Blue arrow=Primary follicle; Green arrow=Secondary follicle; Red arrow=De Graaf follicle. This figure shows that DWE groups have an active proliferation process.

The ovarian morphology shows four different follicles: Primordial, primary, secondary, and de Graaf. The observation and scoring results show that the primordial follicles in the control group are significantly (p≤0.05) higher than other groups (Table-4). Meanwhile, the primary follicles in the DWE3 group were significantly higher than others. The administration of date palm in the DWE2 group significantly increases the number of secondary and de Graaf follicles. The results of the immunohistochemistry staining of the ovarian follicles show that all of the groups are actively proliferating (Figure-2).

**Table 4:** Ovarian morphology of 54 days old laying hens, Lohmann Brown Strain, treated with dates (*Phoenix dactylifera* L.) water extract.

Follicle types	Treatments				

	Control	AGP	DWE1	DWE2	DWE3
Primordial	291.67±9.062^b^	268.33±59.331^ab^	201.67±12.811^ab^	188.67±22.018^a^	202.00±7.234^ab^
Primary	91.00±15.044^a^	116.33±7.513^ab^	138.67±23.947^ab^	111.33±10.682^ab^	160.00±17.156^b^
Secondary	53.33±19.757^a^	90.33±36.005^ab^	169.00±33.779^c^	252.00±32.047^d^	140.33±18.009^bc^
De Graaf	4.00±1.155^a^	4.67±1.202^a^	9.67±1.764^b^	12.00±1.732^b^	9.33±0.333^b^

Control=Mineral water; AGP=Bacitracin 50 mg/kg basal diet+mineral water; DWE1=5% dates water extract; DWE2=10% dates water extract; DWE3=20% dates water extract, dates water extract has been given through drinking water. ^a-d^ Different letters on values in the same row indicate a significant difference (p≤0.05)

### Morphology of lymphoid organ

Table-5 shows that the bursa of Fabricius index in the DWE treatments (DWE1, DWE2, and DWE3) increases compared to control and AGP groups. In addition, DWE1 and DWE2 show higher thymus indexes than other groups. Therefore, the DWE treatments significantly increase the spleen’s white pulp and bursa’s follicle area compared with the control group.

**Table 5:** Lymphoid organs index, white pulp area of spleen and follicle area of bursa of Fabricius of 54 days old laying hens, Lohmann Brown Strain, treated with dates (*Phoenix dactylifera* L.) water extract.

Variables	Treatments				

	Control	AGP	DIW1	DIW2	DIW3
Bursa of Fabricius index	0.11±0.005^a^	0.12±0.006^a^	0.14±0.013^a^	0.15±0.011^a^	0.16±0.009^b^
Thymus index	0.27±0.025^a^	0.33±0.034^ab^	0.41±0.038^b^	0.39±0.041^b^	0.36±0.039^ab^
Spleen index	0.25±0.016^a^	0.36±0.024^ab^	0.42±0.032^c^	0.38±0.031^bc^	0.36±0.019^bc^
White Pulp area of spleen (µm^2^)	5,107.33±50.959^a^	11,138.67±159.144^b^	11,205.33±449.275^b^	17,577.67.±387.968^c^	20,671±93.664^d^
Follicle area of Bursa of Fabricius (µm^2^)	11,111.33±517.283^a^	21,901±871.615^b^	23,882.33±2,659.089^b^	26,920.33±1,831.785^b^	24,593.67±586.075^b^

Control=Mineral water; AGP=Bacitracin 50 mg/kg basal diet+mineral water; DWE1=5% dates water extract; DWE2=10% dates water extract; DWE3=20% dates water extract, dates water extract has been given through drinking water. ^a-d^Different letters on values in the same row indicate a significant difference (p≤0.05)

### Morphometry and laying hens performance

The morphometric measurements of laying hens at the age of 0 and 54 days are shown in Table-6. Laying hens in the DWE2 group have a significant (p≤0.05) back length, chest circumference and depth, and shank length and circumference compared to the control group.

**Table 6:** Body morphometry of 54 days old laying hens, Lohmann Brown Strain, treated with dates (*Phoenix dactylifera* L.) water extract.

Age	Variables	Treatments				

		Control	AGP	DWE1	DWE2	DWE3
0 Day	Chest Depth (mm)	15.53±0.548	15.19±0.476	14.57±0.534	15.23±0.639	15.79±0.614
	Back Length (cm)	5.28±0.065	5.33±0.068	5.20±0.056	5.27±0.104	5.40±0.076
	Chest Circumference (cm)	7.56±0.202	7.89±0.094	7.66±0.169	7.71±0.114	7.59±0.122
	Shank Length (cm)	3.72±0.105	3.69±0.081	3.79±0.087	3.78±0.113	3.74±0.921
	Shank Circumference (cm)	2.01±0.046	2.02±0.071	2.14±0.058	2.16±0.037	2.04±0.054
54 Days	Chest Depth (mm)	52.96±0.353^a^	53.28±0.430^ab^	53.48±0.525^ab^	54.42±0.427^b^	53.12±0.494^ab^
	Back Length (cm)	16.96±0.097^a^	17.61±0.168^b^	17.43±0.092^b^	17.42±0.193^b^	17.52±0.118^b^
	Chest Circumference (cm)	19.26±0.302^a^	19.80±0.315^ab^	19.98±0.396^ab^	20.49±0.336^b^	19.74±0.311^ab^
	Shank Length (cm)	9.13±0.111^a^	9.63±0.092^b^	9.57±0.106^b^	9.70±0.089^b^	9.40±0.094^ab^
	Shank Circumference (cm)	7.17±0.089^a^	7.46±0.103^a^	7.38±0.055^ab^	7.53±0.084^b^	7.37±0.107^ab^

Control=Mineral water; AGP=Bacitracin 50 mg/kg basal diet+mineral water; DWE1=5% dates water extract; DWE2=10% dates water extract; DWE3=20% dates water extract, dates water extract has been given through drinking water. ^a-b^ Different letters on values in the same row indicate a significant difference (p≤0.05)

The effects of DWE on body weight, feed intake, weight gain, and FCR are shown in Table-7. The bodyweight differed significantly (p≤0.05) from 12 to 54 days, with the highest body weight being dominated by DWE2 compared with the control group. In addition, the weight gain on the DWE2 was significantly higher than the control group. At the same time, the feed intake and FCR DWE2 values were significantly lower when compared to the control group.

**Table 7:** Growth performance of 54 days old laying hens, Lohmann Brown Strain, treated with dates (*Phoenix dactylifera* L.) water extract.

Variables	Treatments				
	
Age	Control	AGP	DWE1	DWE2	DWE3
6	59.10±1.370	58.00±0.966	62.50±1.641	63.00±1.680	61.20±1.569
12	103.00±2.216^a^	103.70±2.011^a^	108.90±2.002^ab^	112.60±1.648^b^	108.80±2.133^ab^
18	153.60±2.437^a^	158.40±2.868^a^	169.90±2.002^b^	162.40±3.612^ab^	155.80±3.571^a^
24	208.70±3.252^a^	220.60±5.696^ab^	218.90±5.347^ab^	231.50±5.298^b^	213.10±7.471^a^
30	276.60±5.416^a^	295.70±5.385^ab^	301.90±7.145^b^	303.80±7.298^b^	279.30±8.576^a^
36	306.50±8.128^a^	346.00±6.683^bc^	330.60±8.774^ab^	367.00±9.740^c^	316.30±9.109^a^
42	324.70±10.654^a^	359.00±9.102^ab^	358.60±13.077^ab^	397.70±14.270^b^	366.20±10.197^bc^
48	424.40±13.318^a^	445.70±5.463^ab^	443.50±11.514^ab^	459.80±11.392^b^	434.00±14.484^ab^
54	484.10±11.302^a^	564.60±16.922^bc^	564.40±18.561^bc^	605.20±15.002^c^	533.10±11.824^b^
Feed Intake (g/d)	43.12±0.523^c^	39.96±0.671^ab^	41.80±0.602^bc^	38.09±1.554^a^	42.14±0.874^bc^
Weight Gain (g/d)	48.88±3.952^a^	56.57±10.698^ab^	58.67±13.178^ab^	64.73±12.026^b^	57.82±8.934^ab^
FCR	1.70±0.192^b^	1.41±0.074^ab^	1.38±0.105 ^ab^	1.25±0.064^a^	1.48±0.101^ab^

Control=Mineral water; AGP=Bacitracin 50 mg/kg basal diet+mineral water; DWE1=5% dates water extract; DWE2=10% dates water extract; DWE3=20% dates water extract, dates water extract has been given through drinking water. FCR=Feed conversion ratio. ^a-c^Different letters on values in the same row indicate a significant difference (p≤0.05)

## Discussion

The results of the phytochemical test on DWE showed the presence of secondary metabolites such as 427.41 μg/mL flavonoids, 696.56 μg/mL of tannins, and 33.475 b/v of reducing sugars. Dates contain phenolic components, which inhibit antibacterial activity and bacterial growth through several mechanisms, namely, disrupting bacterial respiration by absorbing metal ions and increasing the concentration of hydrogen peroxide, which causes oxidative stress [21]. Furthermore, there are also potentially known to be potent antioxidants due to bioactive components such as total polyphenols, tannins, flavonoids, and flavanols. Flavonoids promote animal growth through the thalamus-pituitary axis, prevent the formation of free radicals, and increase the body’s resistance to stress [22].

Observations of the small intestine of laying hens showed that DWE2 and DWE3 improve the small intestine morphology. The intestine is part of the digestive system that digests food, and it is the central part directly related to pathogens coming from the environment. Thus, a well-functioning and healthy intestine is essential for poultry. When intestinal function and health are impaired, digestion and absorption of nutrients are not optimal and affect poultry’s health and performance [1]. The performance of DWE starts from the small intestine through the absorption of nutrients.

Furthermore, its addition inhibits the damage to the structure and function of the small intestine through oxidative stress and lipid peroxidation. The small intestine villi are essential in increasing the nutrient absorption ability of the small intestine through the extension of its surface area. Crypt cells migrate from below toward the top of the villi, and it differentiates to form columnar cells, which absorb and take place during the migration process [23].

The flavonoid component can improve the modulation of intestinal absorption, and it protects the digestive tract from damage caused by stress or pathogenic infestation. Furthermore, flavonoids stimulate the mitosis of villi epithelial cells, where the longer or broader villi are associated with the mitotic activity of epithelial cells [19,24]. Our study shows that the flavonoids in DWE have the same effect on increasing villi length and crypt depth. Our phytochemical test shows that dates contain tannins. Kaczmarek *et al*. [25] stated that tannins have antimicrobial activity. This activity can be seen by their ability to pass through the bacterial cell wall up to the internal membrane, interfere with the bacterial metabolism, and causes cell destruction. This action indicates that tannins on dates affected the increases of villi length and crypt depth by their antimicrobial activity.

There are more goblet cells in the jejunum than the duodenum and ileum. The number of goblet cells in the DWE2 group was significantly higher than the control and AGP groups. The area of goblet cells in the DWE2 and DWE3 groups was significantly more extensive than the control and AGP groups. Goblet cells secrete mucus in the digestive tract protects the intestinal membrane from damage caused by enzymes and pathogenic invasion [16]. The results of DWE administration in our study show that the most nutrient absorption occurs in the jejunum than in the duodenum and ileum. The small intestine plays an essential role in the digestion and absorption of nutrients, with the jejunum being the primary location for digesting and absorbing food. This is due to changes in the feed’s shape, often discovered in the jejunum [16,17].

The duodenum, jejunum, and ileum have goblet cells that produce acid mucin, protecting the small intestine from pathogenic bacteria. Mucus contains glycoproteins which are secreted by goblet cells. Furthermore, its formation process increases the mucus capacity of the lumen, thereby preventing pathogens from penetrating the villi. Goblet cells secrete mucus containing glycoproteins to form a gel that acts as a protective barrier for epithelial cells against physical damage through intraluminal substances and inhibits pathogenic bacteria’s invasion; therefore, goblet cells need to be frequently renewed. Flavonoids and tannins are antimicrobial components in dates that prevent goblet cells’ damage [18]. This causes the number and size of goblet cells in the treatment group to be greater than the control group.

Our result of the ovarian morphology in 54 days old laying hens shows that the concentration of DWE in the DWE2 group increases follicle maturation. This is due to the phytoestrogens and flavonoids in dates, which bind to estrogen receptors (ER) and improve follicle maturation. The DWE administration effects are the same as Beazley and Nurmiskaya’s [26] results, which state that female mice offspring are treated with dietary quercetin, which stimulates follicles maturation at the expense of reducing the numbers of the earlier stage follicles (primordial and primary). Quercetin is a flavonoid present in food sources and has an affinity to bind to type I ER, which triggers various estrogenic effects such as cell proliferation and growth [27]. The imbalance between reduce oxidative stress and antioxidants decreases follicular maturation, and thus, the addition of antioxidant supplements is needed to keep its balance. This balance condition prevents cell damage and enhances follicular growth and maturation [28]. The results of DWE administration in our study are in agreement with Shoorei *et al*. [29], which showed that hesperidin increased the PCNA gene expression on ovarian follicle cells. In addition, the PCNA is a 36 kDa protein, which increases during follicle growth and induces survival of the ovarian follicles through DNA replication and repair.

As an avian member, laying hens have central and peripheral lymphoid tissues that play an essential role against pathogens in the body. The increase expresses the index of the lymphoid organ in the sizes of the bursa, thymus, and spleen [30]. From our study results, 10% and 20% DWE as a supplement increases thymus, bursa of Fabricius, and spleen index, which is similar to Chen *et al*. [22] result, that diets supplemented with alfalfa flavonoid promote the size of lymphoid organs such as the thymus, bursa, and spleen. Our study results are also in line with Ramadhanti *et al*. [31], who reported that adding 0.05% *Spirogyra* *jaoensis* ethanolic extract, which contains flavonoids in feed, increases the thymus weight the index of the organ. The positive effect of DWE administration as an antioxidant on the immune response was consistent with an increase in the white pulp area of the spleen and follicle area of the bursa of Fabricius.

Similarly, Attia *et al*. [32] stated that the administration of bee pollen, Propolis, and mannan oligosaccharides as an antioxidant increases bursa’s small and large follicles. The white pulp of the spleen consists of the periarteriolar lymphoid sheath (T-cell area), the adjacent follicles (B-cell area), and the marginal zone (B-cell area) [33]. Furthermore, it was reported that increasing the white pulp area leads to an increase in the immune modulation of laying hens.

Body morphometry is an essential indicator of bone and muscle development. DWE2 supplementation increases back length, chest circumference, chest, shank length, and shank circumference in laying hens aged 54 days old, indicating that DWE has a positive effect on bone and muscle growth of laying hens. The results of our study are in line with Li *et al*. [34], which states that the development of internal organs influences digestion and absorption of nutrients. This is due to the effective deposition of nutrients in muscle and bone tissue. Dates contain phytoestrogens which increase the ability of estrogen to stimulate muscle differentiation. This is due to the binding of phytoestrogens to ERa, which initiates satellite cell marker expression and muscle regeneration [35]. The action mechanism of flavonoids on bone is mediated by the phytoestrogens present in date palm. Furthermore, the bone anabolic reaction induced by flavonoids through osteoblast differentiation on the target molecule is p38 Mitogen-activated protein kinases kinase. Phosphorylation of p38 activates the Wnt signaling pathway, while wnt protein will bind to LRP5/6 (lipoprotein receptor-related protein) Frizzled, which signals the cytosol kinase glycogen synthase kinase-3 β to be phosphorylated. This protein can prevent the phosphorylation of β-catenin. The phosphorylation of β catenin that has been stabilized will be translocated to the nucleus and work together with the transcription factor of lymphoid enhancer factor/T-cell factor to increase the transcription of genes involved in bone differentiation Runx2 gene [36]. In addition, the administration of DWE on laying hens also showed positive results on the pectoral muscle growth, which can be seen from the muscle, fasciculus, and myofiber area. The highest result is in the group administered with 100 mg/mL DWE [37].

The growth performance and body morphometry of laying hens were indirectly related to food digestion and nutrient absorption. Based on Table-5, the results of our study indicate that DWE2 increases body weight and reduces the FCR value, which is similar to El-far *et al*. [10], states that adding 2% and 4% of date seeds to laying hens feed increases body weight and feed efficiency. The flavonoid components found can inhibit pathogenic bacteria and oxidative stress to improve the growth performance of laying hens. Supplemented laying hens with DPK can digest and absorb nutrients efficiently, thereby improving growth performance [9]. Polyphenols and flavonoids act as antibacterial through oxidizing potential, and polyphenols also have the ability of radical scavenging and metal chelating activity [21]. Tannins have antimicrobial activity by complexing with proteins or complexing with polysaccharides. Tannins are also capable bind to the bacteria cell wall; therefore, bacterial growth is inhibited. Tannin also acts as an antioxidant because it has a hydroxyl group that is easily oxidized [38].

Consumption of DWE3 water extract inhibits nutrient absorption; therefore, the DWE3 group has a lower growth performance when compared to the AGP, DWE1, and DWE2 because they have a higher tannin content. Our study results are in line with Rezvani *et al*. [39], in which the administration of pomegranate seed extract negatively influenced growth performance. It is caused by the adverse effects of tannin on birds feeding, and the effects showed on N digestibility, N retention, and productive features. Tannin has an anti-nutritional activity that negatively affects feed intake, nutrient absorption, and growth performance [40].

## Conclusion

It can be concluded that the administration of 10% DWE (100 mg/mL) through drinking water significantly improves the morphology of the small intestine, ovarian follicle, lymphoid organs, and growth performance of laying hens. These findings indicate that DWE can be used as a natural feed additive to improve the performance of laying hens. However, the limitation of this study is that only the tissue morphology and performance were measured, while the expression of gene-level was not explored. Thus, it should be of future interest.

## Authors’ Contributions

HTSSGS, LUA, TIC, and RO: Contributed in the experimentation. HTS and LUA: Wrote and edited the manuscript. HTS, NS, and RDP: Designed the study. All authors read and approved the final manuscript.
